# Supplementary material for: Unraveling the Role of the Liriodendron Thioredoxin (TRX) Gene Family in an Abiotic Stress Response
Source: Plants (Basel). 2024 Jun 17;13(12):1674. doi: 10.3390/plants13121674 (PMC11207409; doi:10.3390/plants13121674)
Supplement: Supplementary file 1 [file plants-13-01674-s001.zip › plants-3019050-supplementary.pdf]

Table S1. The Ka/Ks ratio.

Seq_1	Seq_2	Ka	Ks	Ka_Ks
Lchi00722	Lchi03401	0.328963	1.857507113	0.17709918
Seq_1	Seq_2	Ka	Ks	Ka_Ks
Lchi19206	Lchi21113	0.150407	0.74699186	0.20134996
Seq_1	Seq_2	Ka	Ks	Ka_Ks
Lchi01800	Lchi02097	0.235388	1.366028492	0.172315421

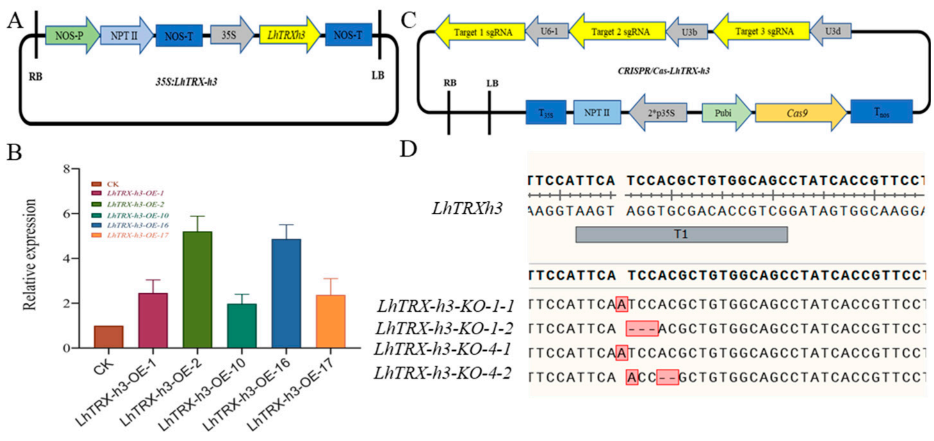

Figure S1. Construction and verification of *LhTRXh3* expression vector. A: *LhTRX-h3* overexpression vector diagram; B: Real-time fluorescence quantification of *LhTRX-h*3-OE， C: *LhTRX-h3*-KO expression vector diagram; D: Sanger sequencing results of *LhTRX-h3*-KO lines.
